# Sunlight exposure during leisure activities and risk of prostate cancer in Montréal, Canada, 2005–2009

**DOI:** 10.1186/1471-2458-14-756

**Published:** 2014-07-28

**Authors:** Jennifer Yu, Jérôme Lavoué, Marie-Élise Parent

**Affiliations:** Department of Environmental and Occupational Health, Université de Montréal, Montreal, Quebec Canada; INRS-Institut Armand-Frappier, Université du Québec, Laval, Québec H7V 1B7 Canada

**Keywords:** Prostate cancer, Sunlight exposure, Leisure, Epidemiology

## Abstract

**Background:**

Prostate cancer (PCa) is the leading cause of cancer in men in many developed countries, but no modifiable risk factors have been identified. A handful of analytical studies have suggested a possible etiological role for sunlight exposure. We report here on the association between leisure-time sunlight exposure during adulthood and PCa risk in the context of a population-based case–control study.

**Methods:**

In all, 1,904 PCa cases were ascertained across Montreal French hospitals between 2005 and 2009. Concurrently, 1,962 population controls, frequency matched to cases by age (±5 years), were selected from the electoral list for French-speakers in Greater Montreal. Interviews elicited the frequency of engagement in any leisure activity during adulthood. This was used to derive cumulative sunlight exposure indices: a cumulative number of leisure activities events entailing sunlight exposure and a cumulative duration of sunlight exposure during leisure activities. Unconditional logistic regression was conducted to yield odds ratios (OR) and 95% confidence intervals (CI) for estimating the association between sunlight exposure indices and PCa risk, adjusting for age, ancestry, family history of PCa, PCa screening, education, solar protection, body mass index and physical activity.

**Results:**

Compared with men in the upper quartile category for the number of sunlight exposure events, men never exposed during leisure time had an OR of 1.32 (95% CI: 0.82-2.14). ORs were 1.11, 0.91 and 1.00 for the first to the third quartiles of exposure, respectively. Similar results were observed for cumulative duration of exposure to sunlight, and by PCa aggressiveness.

**Conclusion:**

These findings provide little evidence of an association between sunlight exposure during leisure-time and PCa risk. Men with no sunlight exposure appeared at somewhat higher risks but none of the estimates achieved statistical significance.

**Electronic supplementary material:**

The online version of this article (doi:10.1186/1471-2458-14-756) contains supplementary material, which is available to authorized users.

## Background

Prostate cancer (PCa) is the leading cause of cancer among men in Canada [1], and in many other developed countries [2]. Despite extensive research, the only clearly established risk factors thus far are increasing age, being of African ancestry, and having a first-degree family history of PCa [2, 3]. However, none of these factors lend themselves to disease prevention. One striking observation comes from migrant studies, which suggest that emigrants tend to acquire the PCa risks of their host countries [4, 5]. This argues for a role of environmental influences, one of which might be sunlight exposure. While the latter has been predominantly linked to an increased risk of skin cancer, a handful of studies have suggested a protective effect of sunlight exposure for other cancers, including breast cancer [6–8].

Epidemiological evidence for a role of sunlight exposure in PCa incidence remains sparse. The majority of previous studies were of ecological design [6, 9–13]. All of these studies, except one [13], suggested a protective effect of sunlight exposure. However, results from the few analytical studies conducted to date, using different sunlight exposure assessment methods, are inconsistent, documenting an inverse association [14–17], no association [18], or a positive association between sunlight exposure and PCa risk [19, 20]. Whether sunlight exposure is actually involved in PCa development remains to be clarified through analytical studies based on strong study designs.

In Quebec, leisure activities represent the main source of sunlight exposure. Less than 8% of workers were reportedly exposed to solar ultraviolet radiation in that province [21]. We present here findings on the association between sunlight exposure, as incurred during outdoor leisure activities across adulthood, and PCa risk, in the context of a population-based case–control study conducted in Montréal, Canada.

## Methods

This analysis is part of PROtEuS (Prostate Cancer & Environment Study), a large scale research project aimed at elucidating the possible role of environmental, occupational, lifestyle, and genetic factors in the development of PCa. This study has been described previously [22].

### Study population

The study was set in Montreal, where over 86% of residents speak French [23]. Cases were actively ascertained through pathology departments in major French hospitals, representing over 80% of all PCa patients diagnosed in the Montreal metropolitan area. Eligible cases were men under 76 years of age, with a first diagnosis of primary prostate adenocarcinoma between September 2005 and December 2009. Concurrently, population controls were randomly selected from Quebec’s permanent French electoral list and frequency matched to cases by age (±5 years). They had no history of PCa. To be eligible, both cases and controls had to be Canadian citizens registered on the provincial electoral list, and to be residents in the Montreal metropolitan area.

Response rates were 79% and 56% among eligible cases and controls, respectively. Reasons for non-participation among cases and controls were refusal (93% and 86%, respectively), unable to trace (2% and 11%, respectively), death with no available proxy (2% and 1%, respectively), language barrier (2% for both), and too sick with no available proxy (1% for controls only) and other (0.1% for controls only). Proxy respondents provided information for 3% and 4% of cases and controls, respectively. The study was approved by the ethics committees of all collaborating institutions (see Additional file 1: Ethics statement), and subjects provided written informed consent.

### Data collection

Face-to-face interviews elicited detailed information on a wide range of socio-demographic, lifestyle and environmental factors. The degree of PCa aggressiveness, as defined by the Gleason score [24], was extracted from pathology reports.

Of relevance to the current analysis, subjects were asked to provide details about prior engagement in any activity or hobby during their leisure time, over their entire adulthood. Specific questions first elicited whether subjects had participated regularly, for at least 6 month, in any of 16 common leisure activities including sports, hobbies and household activities, since they were 18 years of age. Additional questions probed for corresponding information about any other performed hobby or leisure activity not included in the pre-defined list. For each activity reported, subjects were asked when they started and stopped doing the activity, the number of months per year, the frequency per day, week or month. Interruptions and changes in frequency for each activity over the entire adulthood were recorded. The information was used to derive detailed cumulative indices of exposure to sunlight during leisure (see below).

In addition, subjects were asked to self-report their overall frequency of direct exposure to sunlight, separately for leisure time and work time (never, sometimes, and often). Further questions assessed whether subjects used solar protection, such as using sun cream, wearing long sleeves, seeking shelter, etc. when directly exposed to sunlight during leisure as well as at work (never, sometimes, often).

Semi-quantitative assessments of overall physical activity levels during adulthood were elicited from subjects for three types of circumstances, i.e., at home, during leisure-time, and at work (not very active, moderately active, very active). These were combined into a composite index of overall physical activity level for each subject (low, medium, high).

### Sunlight exposure assessment

Sunlight exposure indices were derived from questionnaire information on leisure-time activities. Activities used to assign sunlight exposure were those considered to have been performed outdoors most of the time. The most frequent included sports (walking for exercise, jogging, golf, racket sports, swimming, skiing/skating, cycling, etc.), and gardening and domestic chores (lawn mowing, snow removal, etc.); 108 other types of activities were reported as well (See Additional file 2: Table S1). Some activities, including swimming, could be performed indoor and/or outdoor. Judgment was applied to categorize them as entailing sunlight exposure or not based on the reported frequency and typical circumstances in the Montreal population.

Each leisure-time activity entailing exposure to sunlight is referred to hereafter as an “event”. We calculated, for each subject, two indices of cumulative exposure to sunlight during adulthood. The first index was based on the cumulative number of events (*CEvents*), as follows:
1

where *i* is an individual event, *m* is the total number of different types of events, *Frequency of event* is the number of events per year, *Number of months* is the number of months per year the event occurred, and *Duration in years* is the total number of years of engagement in the event.

The amount of time subjects spent during each leisure activity event could vary both between and within activities, thereby influencing exposure duration per event. To better reflect important differences in duration per event, and to estimate the cumulative number of hours of engagement in activities, we assigned a typical duration to each type of activity based on expected average practices in the study population. For instance, 1 hour was assigned to walking, jogging, swimming and domestic chores requiring physical effort, 2 hours to racket sports, cycling and gardening, 3 hours to skiing or skating and 4 hours to golf. This enabled us to derive a second index based on the cumulative duration of exposure (*CDuration*):
2

Where *Duration of event in hours* is the typical number of hours of engagement attributed to each event.

Some activities were carried out on a seasonal basis. However, information on this was only introduced in the questionnaire one year into the study, by requesting the number of months of participation in each activity over the year. Imputations were thus applied for number of months for the 304 cases and 196 controls with a missing value, reflecting usual patterns from the study base: 12 months per year for walking and domestic chores demanding physical efforts, 6 months per year for jogging, golf, biking and gardening, and 4 months per year for swimming, skiing and skating.

### Statistical analysis

PROtEuS called upon the participation of 1,937 cases and 1,995 controls; subjects with missing information on recreational activities (1 case, 1 control), or on one or several covariates (32 cases, 32 controls) were excluded, leaving 1,904 cases and 1,962 controls for analyses. P-values of respective T tests and Chi-square tests were used to describe the distribution of cases and controls for different variables. A p-value of less than 0.05 designated statistical significance.

Unconditional logistic regression was used to estimate odds ratios (OR) and corresponding 95% confidence intervals (CI) for the association between the two sunlight exposure indices and PCa risk. Subjects were categorized into five sunlight exposure groups, i.e., unexposed subjects, and exposed subjects distributed into quartiles according to the distribution among exposed controls. The upper quartile of exposure was chosen as the reference category because of the small number of unexposed subjects.

Models were adjusted on a set of *a priori* variables, i.e., age (continuous), first-degree family history of PCa (no, yes, don’t know), ancestry (European, African, Asian, Other), and timing of the last PCa screening (2 years earlier, > 2 years earlier, not screened, don’t know). A step-wise forward approach and the Akaike Information Criterion (AIC) [25] were used to identify other covariates to be included in the final models. Variables tested included educational level (elementary, high school, college, university), body mass index (BMI) 2 years before the index date (continuous), solar protection during leisure (never, sometimes, often) and at work (never, sometimes, often), self-reported occupational sunlight exposure (never, sometimes, often) and overall physical activity level (low, medium, high). Solar protection at work and self-reported occupational sunlight exposure were not retained since they did not appreciably improve the fit of the model.

Stratified analyses according to Gleason scores were conducted to evaluate the association between sunlight exposure and PCa according to disease aggressiveness. Non-aggressive PCa was defined by a Gleason score of 6 or lower, or a score of 7 with a primary score of 3, whereas aggressive PCa was defined by a Gleason score of 7 with a primary score of 4, or a score of 8 or higher [26].

Sensitivity analyses were performed by excluding 1) proxy respondents, 2) controls who had not been screened for PCa in the two years before interview, and 3) subjects who were not asked about the number of months per year for each leisure activity. Other analyses evaluated the impact of considering participation to winter leisure activities as non-exposed to the sun, and using the self-reported sunlight exposure during leisure instead of the main exposure indices (*CEvents* and *CDuration*).

Statistical analyses were carried out using the R 2.15.1 statistical software [27, 28].

## Results

Selected characteristics of cases and controls are presented in Table 1. There were slight differences between cases and controls in terms of age, ancestry, BMI, family history of PCa and timing of last PCa screening, while other factors such as education, family income, smoking, alcohol consumption patterns and prevalence of skin cancer (melanoma and non-melanoma skin cancers) did not differ. While the study population was primarily of European ancestry, cases were more often of European or African ancestries, and less often of Asian ancestry, than controls. Cases were twice as likely as controls to have a first-degree family relative with PCa. PCa screening was common in this study population, with nearly all cases and over 76% of controls having been screened in the two years preceding diagnosis or interview. Self-reported overall physical activity levels during adulthood were similar amongst cases and controls.

**Table 1:** **Selected characteristics of 1,904 cases and 1,962 controls, PROtEuS, Montreal, Canada**

Characteristics	Cases		Controls		p-values
**Age in years**, mean (SD)	63.5	(6.8)	64.8	(6.9)	<0.01^d^*
**Ancestry**, n (%)					<0.01^e^*
African	125	(6.6)	86	(4.4)	
Asian	24	(1.3)	71	(3.6)	
European	1681	(88.3)	1674	(85.3)	
Other	74	(3.9)	131	(6.7)	
**Family income in $CAD**, n (%)					0.22^e^
<10,000	55	(2.9)	59	(3.0)	
10,000-19,999	162	(8.5)	176	(9.0)	
20,000-29,999	259	(13.6)	245	(12.5)	
30,000-49,999	442	(23.2)	461	(23.5)	
50,000-79,999	422	(22.2)	406	(20.7)	
80,000-100,000	172	(9.0)	165	(8.4)	
>100,000	252	(13.2)	258	(13.1)	
Unknown	140	(7.4)	192	(9.8)	
**Education**, n (%)					0.18^e^
Primary school or less	432	(22.7)	418	(21.3)	
High school	575	(30.2)	570	(29.1)	
College	310	(16.3)	370	(18.9)	
University	587	(30.8)	604	(30.8)	
**BMI in kg/m** ^**2**^, mean (SD)	26.8	(4.0)	27.2	(4.4)	<0.01^d^*
**Ever smoker** ^**a**^, n (%)					0.46^e^
No	505	(26.5)	501	(25.5)	
Yes	1398	(73.4)	1461	(74.5)	
Unknown	1	(0.1)	0	(0.0)	
**Ever drinker** ^**b**^, n (%)	1697	(89.1)	1738	(88.6)	0.59^e^
**Reported overall physical activity level at work**, n (%)					0.10^e^
Not very active	407	(21.4)	424	(21.6)	
Moderately active	565	(29.7)	639	(32.6)	
Very active	932	(48.9)	899	(45.8)	
**Reported overall physical activity level during leisure time**, n (%)					0.08^e^
Not very active	556	(29.2)	634	(32.3)	
Moderately active	908	(47.7)	931	(47.5)	
Very active	439	(23.1)	396	(20.2)	
Unknown	1	(0.1)	1	(0.1)	
**Reported overall physical activity level at home**, n (%)					0.01^e^*
Not very active	520	(27.3)	592	(30.2)	
Moderately active	945	(49.6)	1001	(51.0)	
Very active	438	(23.0)	369	(18.8)	
Unknown	1	(0.1)	0	(0.0)	
**Had/have skin cancer** ^**c**^, n (%)	62	(3.3)	56	(2.9)	0.47^e^
**First-degree relative with prostate cancer**, n (%)					<0.01^e^*
No	1409	(74.0)	1723	(87.8)	
Yes	445	(23.4)	198	(10.1)	
Unknown	50	(2.6)	41	(2.1)	
**Timing of last prostate cancer screening**, n (%)					<0.01^e^*
Not screened	2	(0.1)	184	(9.4)	
Screened within the last 2 years	1886	(99.1)	1491	(76.0)	
Screened more than 2 years ago	1	(0.1)	234	(11.9)	
Not sure if screened within the last 2 years	15	(0.8)	53	(2.7)	
**Gleason score**, n (%)					
<6/10	15	(0.8)			
6/10	801	(42.1)			
7/10 with primary score of 3	568	(29.8)			
7/10 with primary score of 4	269	(14.1)			
7/10 with unknown primary score	8	(0.4)			
8/10-10/10	243	(12.8)			

^a^Smoked at least 100 cigarettes in lifetime. ^b^Consumed at least one alcohol beverage per month for at least one year. ^c^Includes melanoma and non-melanoma skin cancers. ^d^T test. ^e^Chi-square test. *p-value lower than 0.05.

Table 2 presents sunlight exposure patterns of study subjects. The proportions of cases and controls reporting to have never engaged in any leisure-time activities entailing sunlight exposure during adulthood were 2.9% and 2.7%, respectively. Over half of study subjects reported having engaged in more than three different types of outdoor activities, while the remaining reported between 1 and 3 different types of activities, with similar distributions between cases and controls.

**Table 2:** **Sunlight exposure patterns of 1,904 cases and 1,962 controls, PROtEuS, Montreal, Canada**

Exposure	Cases n (%)		Controls n (%)		p-values
**Individual types of outdoor leisure activities** ^**a**^					
Walking	1206	(75.2)	1246	(63.5)	0.91^b^
Jogging	427	(26.6)	467	(23.8)	0.31^b^
Golf	427	(26.6)	449	(22.9)	0.73^b^
Racket sports	538	(33.5)	527	(26.9)	0.33^b^
Swimming	474	(29.6)	450	(22.9)	0.15^b^
Skiing/skating	893	(55.7)	842	(42.9)	0.01^b^*
Cycling	1108	(69.1)	1037	(52.9)	<0.01^b^*
Gardening	765	(47.7)	799	(40.7)	0.73^b^
Domestic chores demanding physical effort	1562	(97.4)	1605	(81.8)	0.85^b^
Other	367	(19.3)	420	(21.4)	0.10^b^
**Number of different types of outdoor leisure activities** ^**a**^					0.29^c^
0	55	(2.9)	52	(2.7)	
1-3	678	(35.6)	751	(38.3)	
4-11	1171	(61.5)	1159	(59.1)	
**Cumulative number of sunlight exposure events** ***(CEvents)*** ^**a**^					0.30^c^
None: 0	55	(2.9)	52	(2.7)	
Q1: 1–3,210	506	(26.6)	478	(24.4)	
Q2: 3,211-6,642	438	(23.0)	477	(24.3)	
Q3: 6,643-13,679	474	(24.9)	477	(24.3)	
Q4: 13,680-94,886.67	431	(22.6)	478	(24.4)	
**Cumulative number of hours exposed to sunlight** ***(CDuration)*** ^**a**^					0.30^c^
None: 0	55	(2.9)	52	(2.7)	
Q1: 1–4,497	483	(25.4)	478	(24.4)	
Q2: 4,498-9,711	467	(24.5)	477	(24.3)	
Q3: 9,712-18,459	489	(25.7)	476	(24.3)	
Q4: 18,460-103,484	410	(21.5)	479	(24.4)	
**Self-reported sunlight exposure during leisure**					0.03^b^*
Never	105	(5.5)	117	(6.0)	
Sometimes	708	(37.2)	804	(41.0)	
Often	1091	(57.3)	1041	(53.1)	
**Self-reported sunlight exposure at work**					0.12^b^
Never	1238	(65.0)	1318	(67.2)	
Sometimes	345	(18.1)	307	(15.6)	
Often	321	(16.9)	337	(17.2)	
**Use of solar protection during leisure**					0.05^b^
Never	1009	(53.0)	977	(49.8)	
Sometimes	485	(25.5)	502	(25.6)	
Often	410	(21.5)	483	(24.6)	
**Use of solar protection at work**					0.15^b^
Never	1643	(86.3)	1684	(85.8)	
Sometimes	116	(6.1)	101	(5.1)	
Often	145	(7.6)	177	(9.0)	

^a^Exposure between 18 years old and age diagnosis for cases or age at interview for controls. ^b^Chi-square test. ^c^T test. *p-value lower than 0.05.

The median cumulative number of sunlight exposure events, as derived from *CEvents*, was 6,432 events (range = 24 to 94,890) for exposed cases and 6,643 events (range = 24 to 79,190) for exposed controls. The median cumulative duration of leisure-time exposure to sunlight, as derived from *CDuration* was 9,490 hours (range = 24 to 103,500) among exposed cases and 9,712 hours (range = 36 to 86,400) among exposed controls. *CEvents* and *CDuration* were highly correlated with one another (Spearman’s ρ = 0.97).

When subjects were asked to rate their typical frequency of exposure to sunlight, six percent of the cases and controls reported never having been exposed to sunlight during leisure, while about half of the subjects reported to having been often exposed. Almost two-thirds of cases and controls reported having never been exposed to sunlight at work; about 17% of subjects reported having often had sunlight workplace exposure.

About half of sunlight-exposed subjects reported never having used any protection during leisure time; 86% of men indicated having never used protection when exposed at work.

### Association between leisure-time sunlight exposure and PCa risk

Table 3 presents associations between the two indices of sunlight exposure during leisure time and PCa for the whole sample, and by disease aggressiveness. Compared to men who had ever been exposed to sunlight during leisure time activities during adulthood, those who had never been exposed had an OR for PCa of 1.30 (95% CI = 0.83-2.06). In analyses considering quartiles of sunlight exposure, and using the upper quartile as the referent category, there were no statistically significant associations for any of the exposure categories, and PCa. Nor was there evidence of a dose–response pattern. This held true for both cumulative indices, *CEvents* and *CDuration*.

**Table 3:** **ORs (95% CI)** ^**†**^ **for the association between leisure-time sunlight exposure prostate cancer, PROtEuS, Montreal, Canada**

	1,904 controls		All prostate cancers			Non-aggressive prostate cancers^b^			Aggressive prostate cancers^c^		
			1,904 cases and 1,962 controls			1,384 cases and 1,962 controls			512 cases and 1,962 controls		
Sunlight exposure index	n	(%)	n	(%)	OR (95% CI) ^a^	n	(%)	OR (95% CI) ^a^	n	(%)	OR (95% CI) ^a^
***CEvents*** ^**d**^											
None	52	(2.7)	55	(2.9)	1.32 (0.82-2.14)	35	(2.5)	1.29 (0.76-2.20)	19	(3.7)	1.53 (0.79-2.87)
Q1	478	(24.4)	506	(26.6)	1.11 (0.90-1.37)	377	(27.2)	1.12 (0.89-1.41)	128	(25.0)	1.09 (0.79-1.50)
Q2	477	(24.3)	438	(23.0)	0.91 (0.74-1.11)	332	(24.0)	0.94 (0.75-1.17)	105	(20.5)	0.84 (0.61-1.14)
Q3	477	(24.3)	474	(24.9)	1.00 (0.82-1.22)	324	(23.4)	0.92 (0.73-1.15)	146	(28.5)	1.19 (0.89-1.59)
Q4	478	(24.4)	431	(22.6)	1.00 (reference)	316	(22.8)	1.00 (reference)	114	(22.3)	1.00 (reference)
					*p-trend* = 0.56			*p-trend* = 0.57			*p-trend* = 0.58
***CDuration*** ^**e**^											
None	52	(2.7)	55	(2.9)	1.38 (0.86-2.23)	35	(2.5)	1.41 (0.83-2.41)	19	(3.7)	1.41 (0.73-2.65)
Q1	478	(24.4)	483	(25.4)	1.10 (0.89-1.37)	363	(26.2)	1.18 (0.93-1.49)	118	(23.0)	0.92 (0.67-1.26)
Q2	477	(24.3)	467	(24.5)	0.99 (0.81-1.22)	333	(24.1)	1.00 (0.79-1.25)	134	(26.2)	0.99 (0.73-1.33)
Q3	476	(24.3)	489	(25.7)	1.13 (0.92-1.39)	366	(26.4)	1.20 (0.96-1.50)	119	(23.2)	0.95 (0.70-1.28)
Q4	479	(24.4)	410	(21.5)	1.00 (reference)	287	(20.7)	1.00 (reference)	122	(23.8)	1.00 (reference)
					*p-trend* = 0.38			*p-trend* = 0.30			*p-trend* = 0.66

^†^Odds ratios and 95% confidence intervals. ^a^Adjusted for age, first-degree family history of prostate cancer, ancestry, timing of last prostate cancer screening, education, BMI, solar protection during leisure and overall physical activity level. ^b^Prostate cancer cases with a Gleason score of 6 or lower or of 7 with a primary score of 3. ^c^Prostate cancer cases with Gleason score of 7 with a primary score of 4 or 8 or higher. ^d^Cumulative number of events entailing sunlight exposure from age 18 years to age at diagnosis for cases or age at interview for controls (levels: none: 0 event, Q1: 1 to 3,210 events, Q2: 3,211 to 6,642 events, Q3: 6,643 to 13,679 events, Q4: 13,680 to 94,886.67 events). ^e^Cumulative duration of leisure-time exposure to sunlight from age 18 years to age at diagnosis for cases or age at interview for controls (levels: none: 0 hour, Q1: 1 to 4,497 hours, Q2: 4,498 to 9,711 hours, Q3: 9,712 to 18,459 hours, Q4: 18,460 to 103,484 hours).

Similar results were obtained upon stratifying by PCa aggressiveness (Table 3). In addition, all five sensitivity analyses (Table 4) showed results comparable with those from the main analyses.

**Table 4:** **Sensitivity analyses for the association between leisure-time sunlight exposure and prostate cancer, PROtEuS, Montreal, Canada**

	Cases		Controls		
Sensitivity analysis	n	(%)	n	(%)	OR (95% CI)^a^
**Excluding proxy respondents**	1854	(100)	1889	(100)	
***CEvents*** ^**b**^					
None: 0 event	55	(3.0)	46	(2.4)	1.45 (0.89-2.38)
Q1: 1 to 3,210 events	490	(26.4)	456	(24.1)	1.13 (0.91-1.40)
Q2: 3,211 to 6,642 events	431	(23.2)	463	(24.5)	0.94 (0.76-1.16)
Q3: 6,643 to 13,679 events	464	(25.0)	460	(24.4)	1.01 (0.82-1.24)
Q4: 13,680 to 94,886.67 events	414	(22.3)	464	(24.6)	1.00 (reference)
					*p-trend* =0.52
***CDuration*** ^**c**^					
None: 0 hour	55	(3.0)	46	(2.4)	1.52 (0.94-2.50)
Q1: 1 to 4,497 hours	466	(25.1)	457	(24.2)	1.14 (0.91-1.42)
Q2: 4,498 to 9,711 hours	459	(24.8)	465	(24.6)	1.01 (0.82-1.25)
Q3: 9,712 to 18,459 hours	480	(25.9)	457	(24.2)	1.18 (0.96-1.46)
Q4: 18,460 to 103,484 hours	394	(21.3)	464	(24.6)	1.00 (reference)
					*p-trend* =0.35
**Excluding controls not screened for prostate cancer**	1904	(100)	1491	(100)	
***CEvents*** ^**b**^					
None: 0 event	55	(2.9)	34	(2.3)	1.39 (0.86-2.27)
Q1: 1 to 3,210 events	506	(26.6)	336	(22.5)	1.12 (0.91-1.39)
Q2: 3,211 to 6,642 events	438	(23.0)	373	(25.0)	0.88 (0.72-1.09)
Q3: 6,643 to 13,679 events	474	(24.9)	374	(25.1)	1.03 (0.84-1.26)
Q4: 13,680 to 94,886.67 events	431	(22.6)	374	(25.1)	1.00 (reference)
					*p-trend* =0.56
***CDuration*** ^**c**^					
None: 0 hour	55	(2.9)	34	(2.3)	1.49 (0.92-2.45)
Q1: 1 to 4,497 hours	483	(25.4)	339	(22.7)	1.18 (0.95-1.46)
Q2: 4,498 to 9,711 hours	467	(24.5)	370	(24.8)	1.00 (0.81-1.23)
Q3: 9,712 to 18,459 hours	489	(25.7)	365	(24.5)	1.19 (0.97-1.46)
Q4: 18,460 to 103,484 hours	410	(21.5)	383	(25.7)	1.00 (reference)
					*p-trend* =0.32
**Excluding subjects without information for number of months per year of leisure activities**	1611	(100)	1766	(100)	
***CEvents*** ^**b**^					
None: 0 event	55	(3.4)	52	(2.9)	1.48 (0.92-2.41)
Q1: 1 to 3,210 events	428	(26.6)	428	(24.2)	1.17 (0.93-1.47)
Q2: 3,211 to 6,642 events	381	(23.6)	434	(24.6)	0.97 (0.78-1.21)
Q3: 6,643 to 13,679 events	396	(24.6)	414	(23.4)	1.07 (0.86-1.32)
Q4: 13,680 to 94,886.67 events	351	(21.8)	438	(24.8)	1.00 (reference)
					*p-trend* =0.41
***CDuration*** ^**c**^					
None: 0 hour	55	(3.4)	52	(2.9)	1.55 (0.96-2.52)
Q1: 1 to 4,497 hours	397	(24.6)	429	(24.3)	1.12 (0.89-1.42)
Q2: 4,498 to 9,711 hours	407	(25.3)	428	(24.2)	1.09 (0.87-1.36)
Q3: 9,712 to 18,459 hours	426	(26.4)	428	(24.2)	1.24 (0.99-1.54)
Q4: 18,460 to 103,484 hours	326	(20.2)	429	(24.3)	1.00 (reference)
					*p-trend* =0.30
**Considering winter leisure activities considered as non-exposed**	1904	(100)	1962	(100)	
***CEvents*** ^**b**^					
None: 0 event	60	(3.2)	54	(2.8)	1.47 (0.93-2.35)
Q1: 1 to 2,173 events	499	(26.2)	477	(24.3)	1.19 (0.96-1.48)
Q2: 2,174 to 4,560 events	430	(22.6)	477	(24.3)	0.97 (0.79-1.20)
Q3: 4,561 to 9,103 events	525	(27.6)	477	(24.3)	1.24 (1.01-1.52)
Q4: 9,104 to 52,991.5 events	390	(20.5)	477	(24.3)	1.00 (reference)
					*p-trend* =0.34
***CDuration*** ^**c**^					
None: 0 hour	60	(3.2)	54	(2.8)	1.38 (0.87-2.20)
Q1: 1 to 2,987 hours	455	(23.9)	477	(24.3)	1.03 (0.83-1.28)
Q2: 2,988 to 6,790 hours	474	(24.9)	477	(24.3)	1.01 (0.82-1.24)
Q3: 6,791 to 12,479 hours	492	(25.8)	476	(24.3)	1.14 (0.93-1.40)
Q4: 12,480 to 94,933 hours	423	(22.2)	478	(24.4)	1.00 (reference)
					*p-trend* =0.46
**Using self-reported recreational sunlight exposure**	1904	(100)	1962	(100)	
None	105	(5.5)	117	(6.0)	0.98 (0.71-1.37)
Sometimes	708	(37.2)	804	(41.0)	0.91 (0.79-1.06)
Often	1091	(57.3)	1041	(53.1)	1.00 (reference)

^a^Odds ratio (OR), 95% confidence interval (CI). Models adjusted for age, first-degree family history of prostate cancer, ancestry, timing of last prostate cancer screening, education, BMI, solar protection during leisure and overall physical activity level. Analyses excluding controls not screened for prostate cancer in the previous 2 years are not adjusted for prostate cancer screening. ^b^Cumulative number of events entailing sunlight exposure from age 18 years. ^c^Cumulative duration of leisure-time exposure to sunlight from age 18 years.

## Discussion

There was little evidence of an association between sunlight exposure during leisure-time and PCa risk in our data. Men never exposed to sunlight during leisure-time appeared to be at slightly higher risk of PCa than those who had been exposed at some point during adulthood, but none of the estimates achieved statistical significance.

The epidemiological evidence available to date on the association between sunlight exposure and PCa risk remains contentious. Some previous studies have looked at this association in terms of PCa mortality [29–34]. Since the latter can reflect both survival and etiology, findings from these are not expected to necessarily align with our own, which focuses on PCa incidence.

Of studies assessing the risk of incident PCa [6, 9–20, 35], many were in line with a protective effect of sunlight. However, the majority used an ecological approach, with confounding remaining of major concern [36], especially when studying sunlight exposure and cancer risk [13].

To our knowledge, only eight analytical studies of incident PCa have been conducted to date on this issue [14–20, 35]. Study design, size and sunlight assessment protocols have varied widely. Five of these support a protective effect of sunlight. Two were case–control studies, one [14] assessing exposure to sunlight residential, work and recreational settings as part of interviews (450 cases, 455 controls), the other [35] assessing cumulative sunlight exposure per year and sunbathing frequency (453 cases, 312 controls). Three cohort studies also reported a protective effect of sunlight. The first was based on 153 PCa cases, and estimated residential sunlight ambient levels [15]. The second, including 161 PCa cases, assessed solar radiation levels at birth address and reports of sunlight exposure during leisure and at work [16]. More recently, a third and much larger cohort study accrued over 21,000 incident PCa cases during the period of follow-up [17]. This investigation assessed ambient ultraviolet radiation levels at the residence of subjects at baseline. However, individual information on time spent outdoors, which can vary greatly between individuals and over time, was not available.

Another large investigation, a nested case–control study of 1,020 cases and 5,044 controls conducted in the UK, observed no association overall between reported time spent outside during childhood and adulthood, and PCa risk [18]. An inverse relationship was observed between PCa and intense sun exposure 2 years prior to the index date (all cancers), while time spent outside was associated with higher risks of advanced PCa.

Finally, two case–control studies documented an excess risk of PCa among individuals who had spent the greatest number of hours outdoor either recently [19], or at the ages of 30 and 50 years [20]. One [19] was conducted in Singapore (240 cases, 268 controls) while the other [20], in New South Wales, Australia (1084 cases and 234 controls). In both studies, positive associations were apparent for all cancers, as well as for aggressive ones [19]. It has been brought forward that a U-shaped relationship might reconcile apparent contradictions across studies, i.e., inverse or positive associations between sunlight exposure and PCa observed in low or high solar UV environments, respectively [20]. In our study, none of the sunlight exposure levels showed statistically significant associations with PCa and confidence intervals overlapped between exposure categories. However, men in the middle category tended to be at lesser risk of PCa compared to those in other categories. Tests assessing the linearity of the exposure variables or the presence of a U-shape association were negative (data not shown).

Previous studies present methodological differences with our own. One salient difference across previous investigations relates to the sunlight exposure assessment methods applied. Geographical-based sunlight exposure indices were used in ecological studies [6, 9–12] as well as in some analytical studies [14–17]. The downside of such approaches is that ambient solar levels are assumed to reflect individual exposures, which may result in important misclassification of exposure. Individual-based exposure assessment methods used in previous studies include a self-reported recreational sun exposure level [16], a sun exposure index based on skin pigmentation [14], lifetime or adulthood frequencies of outdoor leisure activities in hours per week [14, 19] and a sum of hours of sunlight exposure during summer weekends at two age points [20]. Although our sunlight exposure assessment may not be as precise as that used in some of the aforementioned studies, it was based on detailed questionnaire data eliciting participation in outdoor leisure activities. Changes in engagement in the different activities over the years were factored in, so were variations across seasons for most study subjects. However, we did not collect information on acute sunlight exposures (e.g. sunbathing), on sunlight exposure while traveling to work or elsewhere, or on the specific number of hours involved in each outdoor activity. Nevertheless, when we applied typical leisure activity event durations to differentiate between typically shorter and longer exposure events it had little impact on risk estimates as compared to the cumulative number of events. We had information on the reported overall sunlight exposure during work-time. Albeit resulting in a crude exposure index, workplace exposure had relatively low prevalence in our population and did not influence the association between leisure-time exposure and PCa.

Our study was based on the population living in Montreal, Canada, at a latitude entailing low solar radiation intensity [37]. It may be that our findings are not readily comparable with those observed among subjects living in different latitudes. Another difference relates to the ancestries represented in the different study populations. Our study predominantly included subjects of European ancestry, whereas, for instance, the Singapore study focused on Asians, a population known to have lower risks of PCa [38].

As is the case for any study evaluating risks or benefits associated with sunlight, exposure misclassification likely occurred to some extent in our data. However, it is believed to have been largely non-differential, which tends to result in conservative estimates [39]. Questions on leisure activities were primarily formulated to assess energy expenditure and subjective judgment needed to be applied in a number of occasions when assigning them as indoor or outdoor activities. Physical activity is potentially related with PCa [40, 41], and we adjusted for it in our analyses.

Reporting bias based on case–control status was possible, but unlikely. Sunlight exposure was not the primary focus of the PROtEuS study. Moreover, there is no widespread belief in the population that sunlight exposure is associated with PCa.

The vitamin D mechanism is most often called upon to explain a possible protective effect of sunlight exposure on PCa. Different pathways may be involved, such as decreased cell proliferation, cell cycle regulation, cell invasiveness and angiogenesis and increased cellular differentiation and apoptosis [42, 43]. Although the evidence for an anti-cancer mechanism of vitamin D is strongly suggested by biological studies, it has not been consistently supported by epidemiological findings [44]. It is thus possible that other mechanisms exist between sunlight exposure and PCa. One such mechanism, less documented in sunlight exposure studies, could be UV-induced nitric oxide [45]. At high concentrations, inhibitions of prostate cancer cell proliferation, of metastases and of epithelial-mesenchymal transition have been observed, and at low concentrations angiogenesis is promoted.

Should a relation between sunlight and PCa truly exist, and should this association involve vitamin D synthesis, then a number of factors would need to be considered for valid exposure assessment, such as skin coverage by clothing, skin type, geographical location and meteorological conditions [37]. First, the amount of skin exposed to the sun can determine the rate of vitamin synthesis and darker skins tend to allow for less vitamin D production [46]. However, previous data indicate that after a certain threshold, a greater exposed surface and a higher dose of UVB will not significantly increase vitamin D levels [47]. This could be explained by the negative feedback loop triggered by high levels of vitamin D, which involves its inactivation by the CYP24A1 hydroxylase [42]. Second, the amount of solar radiation received has been shown to differ between geographical regions, even when doing the same activity for the same amount of time [48]. Third, meteorological conditions such as the presence of direct, diffuse and reflected radiations, the time of the day and the atmospheric conditions [49] also affect the amount of solar radiation received. In addition, diet and supplementation play a role in the level of serum vitamin D, especially in the winter months when, at northern latitudes, no important vitamin D synthesis is triggered [50]. Information for these factors was not available for our study, so each recreational sunlight event was assumed here to be equal in terms of sunlight exposure dose.

Our study presents several important strengths. This is the largest study to date to assess the association between sunlight exposure, using an assessment protocol that takes into account individual sunlight-related behavior, and PCa. Moreover, the exposure assessment covered the entire adulthood period, something rarely achieved in the past. Cases were histologically confirmed, and information was collected as part of face-to-face interviews. Participation rates in the study were relatively good, compared to those often observed in similar studies. We were able to compare socio-demographic characteristics of eligible men who declined to participate in the study, to those of study subjects; only slight differences were observed, suggesting that strong selection bias was not at play. We collected information on a wide range of potential confounders and considered these in our analyses. This included the use of solar protection, which few studies have been able to take into account [18, 20].

## Conclusion

Overall, there was little evidence in our data of an association between leisure-time exposure to sunlight during adulthood, and PCa development. Men never exposed to sunlight tended to show somewhat higher risks than those highly exposed, but confidence intervals included the null value and there was no dose–response pattern. These observations applied to both non-aggressive and aggressive PCa. Further studies of PCa based on refined sunlight exposure assessment protocols taking into account individual variations should be undertaken. Should sunlight exposure be shown to be linked to PCa risk, either negatively or positively, such a finding would be of high public health importance.

## Electronic supplementary material

Additional file 1: **Ethics statement.** (DOCX 15 KB)

Additional file 2: Table S1: List of entries of leisure activities not predefined in the general questionnaire, PROtEuS, Montreal, Canada. (DOCX 26 KB)

## Abbreviations

AIC: Akaike information criterion
BMI: Body mass index
*CDuration*: Cumulative duration of sunlight exposure during leisure activities during adulthood
*CEvents*: Cumulative number of leisure activity events entailing sunlight exposure during adulthood
CI: Confidence intervals
OR: Odds ratios
PCa: Prostate cancer
PROtEuS: Prostate cancer & environment study
SD: Standard deviation.
