# Supplementary material for: Sunlight exposure during leisure activities and risk of prostate cancer in Montréal, Canada, 2005–2009
Source: BMC Public Health. 2014 Jul 28;14:756. doi: 10.1186/1471-2458-14-756 (PMC4122789; doi:10.1186/1471-2458-14-756)
Supplement: Supplementary file 1 — Additional file 1: Ethics statement. (DOCX 15 KB) [file 12889_2014_6886_MOESM1_ESM.docx]

## Ethics statement

This study has been approved by the following ethics committees: Research Ethics Committee of the Centre de Recherche du Centre Hospitalier de l’Université de Montréal (hospitals: Notre-Dame, St-Luc, Hôtel-Dieu de Montréal), Research Ethics Committee of the Maisonneuve-Rosemont Hospital, the Research Ethics Committee of Jean-Talon Hospital, the Research Ethics Committee of the Fleury Hospital and the Research Ethics Committee of the Centre de Santé et de Services Sociaux Champlain-Charles-LeMoyne. In addition it was approved by the Comité d’Éthique en Recherche avec des êtres humains (CER) from the Institut National de la Recherche Scientifique (INRS) and the Comité d’éthique de la recherché en santé (CERES) from the Université de Montréal. All participants provided written informed consent.
