# Supplementary material for: Sunlight exposure during leisure activities and risk of prostate cancer in Montréal, Canada, 2005–2009
Source: BMC Public Health. 2014 Jul 28;14:756. doi: 10.1186/1471-2458-14-756 (PMC4122789; doi:10.1186/1471-2458-14-756)
Supplement: Supplementary file 2 — Additional file 2: Table S1: List of entries of leisure activities not predefined in the general questionnaire, PROtEuS, Montreal, Canada. (DOCX 26 KB) [file 12889_2014_6886_MOESM2_ESM.docx]

| **Supplementary table 1 – List of entries of all leisure activities not predefined in the general questionnaire, PROtEuS, Montreal, Canada** | | |
| --- | --- | --- |
| **French or reported name^a^** | **English name** | **Number of entries** |
| alpinisme | mountaineering | 7 |
| athlétisme | athletics | 2 |
| aviation | aviation | 1 |
| aviron | rowing | 4 |
| ballon-panier | basketball | 1 |
| baseball | baseball | 109 |
| baseball (entraineur) | baseball (coach) | 1 |
| baseball (instructeur) | baseball (coach) | 1 |
| basketball | basketball | 34 |
| bateau | boat | 2 |
| bateau dragon | dragon boat | 1 |
| bicyclette | cycling | 25 |
| camping | camping | 2 |
| canoe kayak | ark, kayak | 1 |
| canot | ark | 19 |
| canot camping | ark, camping | 1 |
| canot de course | ark racing | 1 |
| canot descente | ark | 2 |
| chasse | hunting | 42 |
| chasse et pêche | hunting and fishing | 4 |
| compétition équestre | equestrian competition | 1 |
| construction résidentielle | house building | 1 |
| coupe de bois | wood cutting | 7 |
| course traineau chien | dog sleigh | 1 |
| cricket | cricket | 6 |
| deck hockey | street hockey | 1 |
| diverses activites extérieures (animateur scout) | diverse outdoor activities (scout animator) | 2 |
| équitation | horse riding | 20 |
| équitation (entraineur) | horse riding (coach) | 1 |
| escalade | climbing | 5 |
| exploitation forêt | forest exploitation | 1 |
| fastball | fastball | 4 |
| fers | horseshoes | 2 |
| football | football | 53 |
| football (arbitre) | football (referee) | 1 |
| football américain | American football | 17 |
| football et soccer | football and soccer | 2 |
| football touch | football | 1 |
| golf | golf | 22 |
| hiking | hiking | 6 |
| hockey bottine | street hockey | 2 |
| montée à cheval | horse riding | 1 |
| jardinage | gardening | 1 |
| jogging / course | jogging | 4 |
| kayak | kayak | 8 |
| kayak de mer | kayak | 2 |
| kayak et pédalo | kayak and pedalo | 1 |
| lawn bowling | lawn bowling | 1 |
| marche | walking | 25 |
| marche en montagne | hiking | 1 |
| marche rapide | fast walking | 1 |
| moto | motorcycle | 2 |
| motocross | motocross | 2 |
| motoneige | snowmobile | 2 |
| natation | swimming | 10 |
| parachutisme | skydiving | 3 |
| patinage ou ski | skating or skiing | 4 |
| patins à roues alignees | roller-skating | 20 |
| pêche | fishing | 52 |
| pêche au saumon | salmon fishing | 1 |
| pêche sur glace | ice fishing | 2 |
| pétanque | bowls | 21 |
| pilote avion sportif | aircraft pilot | 1 |
| pilote planneur | gliding | 1 |
| planche à neige | snowboarding | 1 |
| planche à voile | windsurfing | 17 |
| plongée | diving | 6 |
| plongée en apnée | snorkelling | 1 |
| plongée sous-marine | scuba diving | 14 |
| randonnée pédestre | hiking | 3 |
| raquette | snowshoeing | 26 |
| raquette à neige | snowshoeing | 5 |
| roller skating | roller-skating | 1 |
| rollerblade | roller-blading | 5 |
| rowing (club) | rowing | 1 |
| rugby | rugby | 6 |
| sailboard | sailboard | 1 |
| sailing | sailing | 1 |
| saut à la perche | pole vault | 1 |
| scuba diving | scuba diving | 1 |
| shuffleboard | shuffleboard | 2 |
| ski | skiing | 4 |
| ski alpin | alpine skiing | 1 |
| ski aquatique | waterskiing | 2 |
| ski de fond | cross-country skiing | 4 |
| ski nautique | waterskiing | 16 |
| snorkeling | snorkelling | 1 |
| snowshoeing | snowshoeing | 1 |
| soccer | soccer | 290 |
| soccer (arbitre et entraineur) | soccer (referee and coach) | 1 |
| soccer (entraineur) | soccer (coach) | 4 |
| softball | softball | 26 |
| sports de raquette | racket sports | 4 |
| tennis | tennis | 2 |
| tir à l’arc | archery | 7 |
| touch football | touch football | 3 |
| track & field | track and field | 1 |
| trappage | hunting | 1 |
| travaux domestiques^a^ | domestic work | 24 |
| viticulture | viticulture | 1 |
| voile | sailing | 39 |
| voile catamaran | catamaran sailing | 1 |
| voile dériveur | dinghy sailing | 1 |
| voile régate | regatta sailing | 2 |
| voile sportive | sailing | 1 |
| windsurf | windsurfing | 1 |
| ^a^ Designates domestic work involving physical effort, e.g. lawn mowing, snow shovelling. | | |
